# FEASIBILITY OF MOBILE HEALTH FOR LOW-INCOME MINORITY HISPANIC PATIENTS WITH A STROKE

**DOI:** 10.1093/geroni/igz038.1215

**Published:** 2019-11-08

**Authors:** Stuti Dang, Kasra Sarhadi, Sonjia Kenya, Chuanhui Dong, Natalie Ferras, Jose Romano, Olveen Carrasquillo

**Affiliations:** 1 Miami Veterans Affairs Healthcare System- GRECC, Miami, Florida, United States; 2 University of Miami Miller School of Medicine, Miami, Florida, United States

## Abstract

Stroke is a leading cause of death and functional impairment that disproportionately impacts Hispanics. Several studies have supported the feasibility of mobile health interventions (mHealth) to provide health monitoring and patient education for improving chronic disease management, but none have focused on Latino stroke patients. The Hispanic Secondary Stroke Prevention Initiative is a randomized study of 200 stroke patients designed to evaluate the impact of a 12-month multi-modal Community Health Worker (CHW) and mHealth intervention on blood pressure control. Eligible participants were Latinos who experienced a mild-moderate stroke within the last five years. The CHW component included home visits, telephone calls, and daily text messages to obtain home blood pressure readings and provide patient navigation and health education. Feasibility was defined as the proportion of patients that responded to at least half the messages. Pre-post paired t-tests assessed improvements in question accuracy while correlation coefficients highlighted improvements in response rates. Among the 65 participants randomized to the intervention, the response rate was as follows: 37% - >50% response, 21% - 25-50%, 19% - 10-25%, and 23% - <10%, This finding suggests that mHealth interventions may be challenging in this population. However, the proportion of questions answered correctly increased from 63% to 84% in the intervention period’s last two months (p<0.05). There was a positive correlation between increased response rates and response accuracy to patient education assessments (r=0.82, p<0.05). These improvements in health knowledge suggest that a subset of patients may benefit from mHealth interventions, and the benefit correlates with use.

